# Supplementary material for: Redirect Tropism of Fowl Adenovirus 4 Vector by Modifying Fiber2 with Variable Domain of Heavy-Chain Antibody
Source: Genes (Basel). 2024 Apr 8;15(4):467. doi: 10.3390/genes15040467 (PMC11049955; doi:10.3390/genes15040467)
Supplement: Supplementary file 1 [file genes-15-00467-s001.zip › genes-2928846-supplementary.pdf]

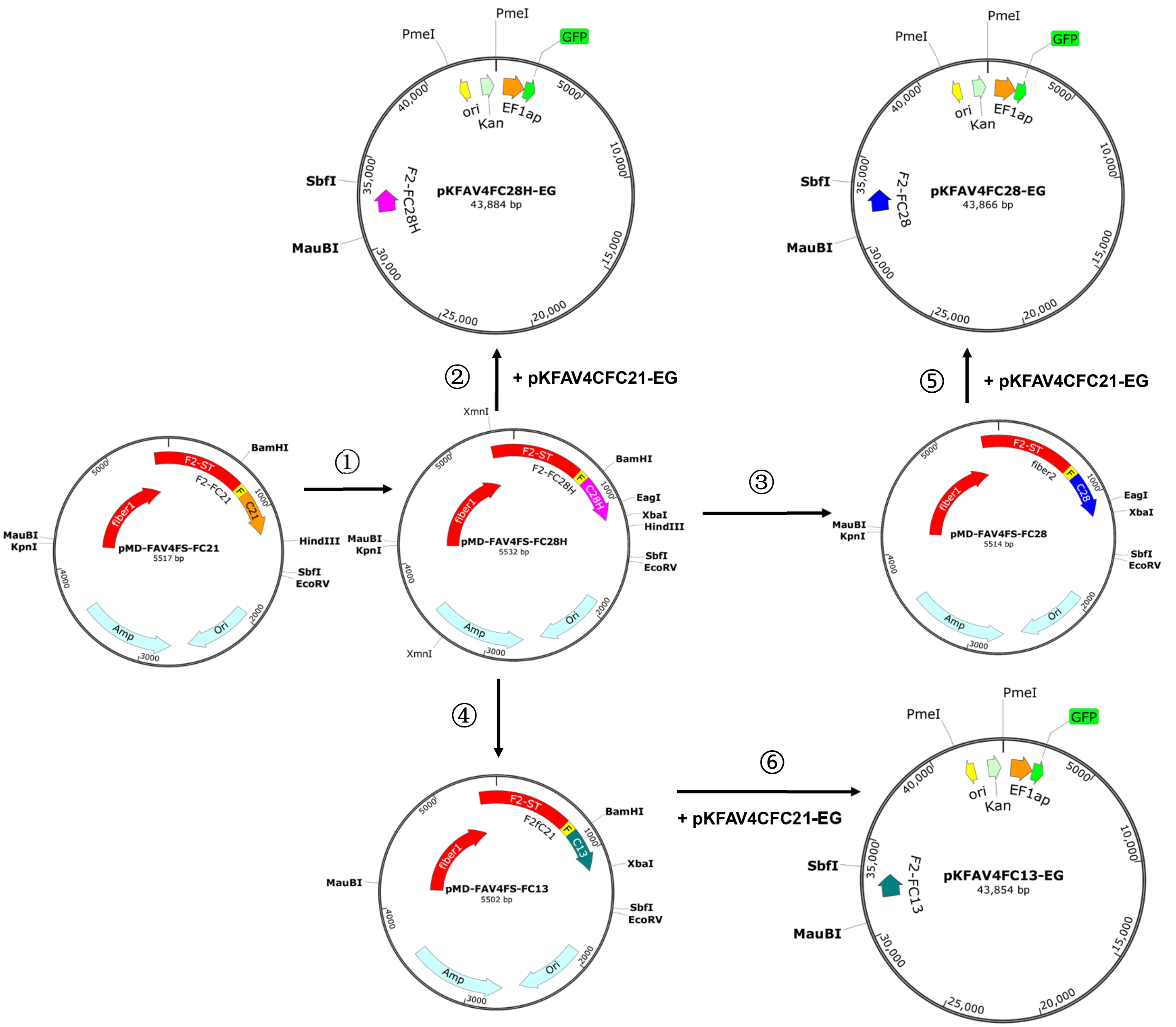

Figure S1. Construction of VHH-incorporated FAdV-4 adenoviral plasmids.
① BamHI/HindIII region in pMD-FAV4FS-FC21 was replaced with the synthesized C28H fragment by restriction-assembly to generate pMD-FAV4FS-FC28H. ② pMD-FAV4FS-FC28H was digested with KpnI/EcoRV, pKFAV4CFC21-EG was digested with MauBI/SbfI, the products were mixed and Gibson assembly was performed to generate pKFAV4FC28H-EG. ③ To further remove the 6×His tag in modified fiber2, the se-quence between EagI/XbaI was mutated by overlap extension PCR, and modified plasmid of pMD-FAV4FS-FC28 was generated by restriction-assembly. ④ Fragment between BamHI/XbaI site in pMD-FAV4FS-FC21 was replaced with the synthesized C13 fragment by restriction-assembly to generate pMD-FAV4FS-FC13. ⑤ ⑥ Similarly, modified fiber2 could be transfer to pKFAV4CFC21-EG by restriction-assembly.
C13, C21 and C28: VHHs against human CD16A; CMVp: CMV promoter; EF1ap: human EF1a promoter; Coiled coil: the 12th-13th coiled coil segments from fibritin of phage T4; F2-ST: FAdV-4 fiber2 shaft and tail.

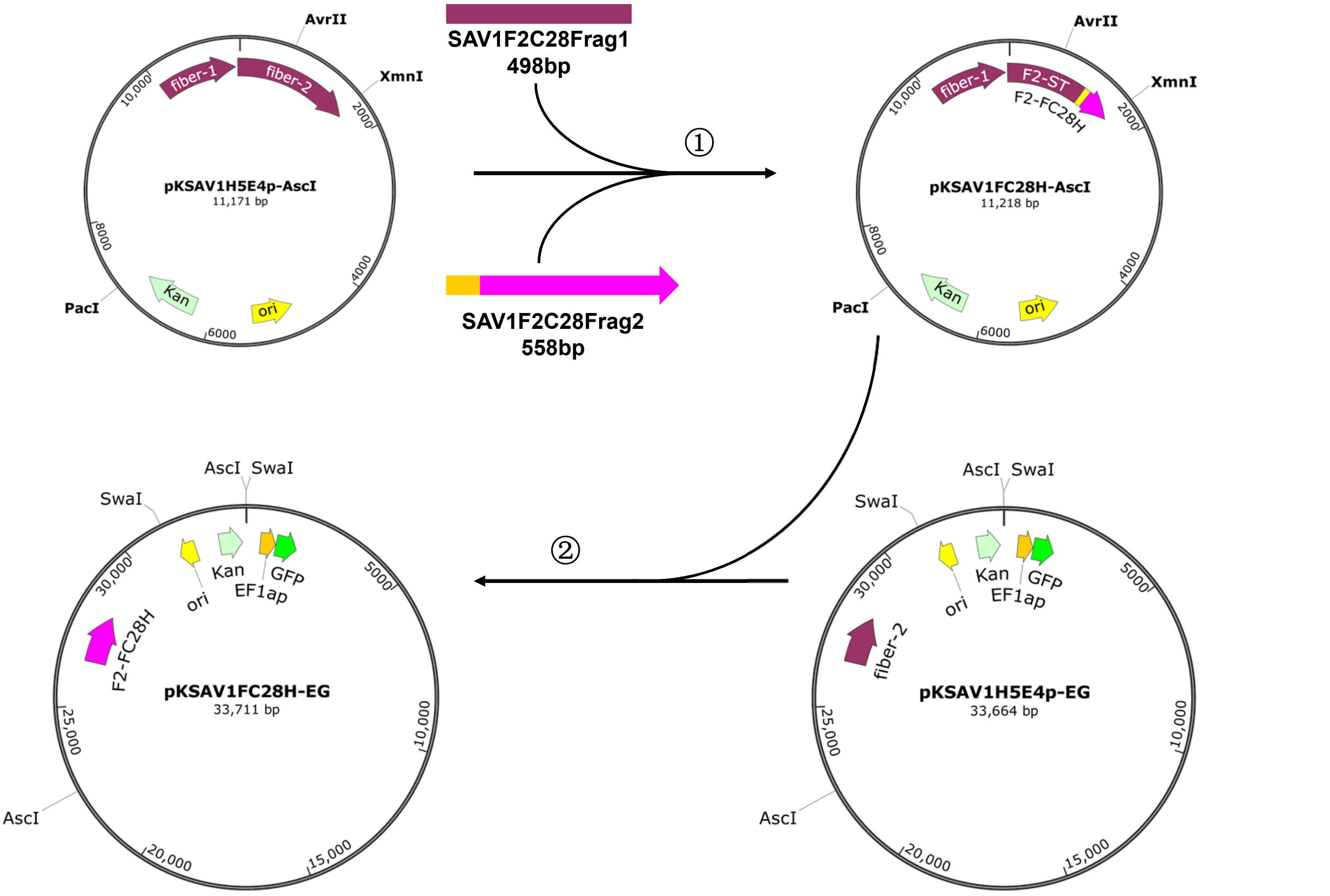

Figure S2. Construction of pKSAV1FC28H-EG adenoviral plasmid.
① For fiber modification in SAdV-1 vectors, overlap extension PCR was performed to fuse several PCR products together to generate a DNA fragment FC28H, which consists of SAV1F2C28Frag1, encoding partial SAdV-1 fiber2 shaft and tail, SAV1F2C28Frag2, encoding VHH of C28 against human CD16A, sequence surrounding AvrII site and sequence surrounding XmnI site in pKSAV1H5E4p-AscI plasmid. Fragment between AvrII/XmnI sites in pKSAV1H5E4p-AscI was replaced with the synthesized DNA C28H by restriction-assembly to generate pKSAV1FC28H-AscI.
② pKSAV1FC28H-AscI was digested with PacI, pKSAV1H5E4p-EG was digested with AscI, the products were mixed and Gibson assembly was performed to generate pKSAV1FC28H-EG. The primer sequences and related information for PCR was summarized in Table 1.
C28: VHH against human CD16A; EF1ap: human EF1a promoter; Coiled coil: the 12th-13th coiled coil segments from fibritin of phage T4; F2-ST: SAdV-1 fiber2 shaft and tail.

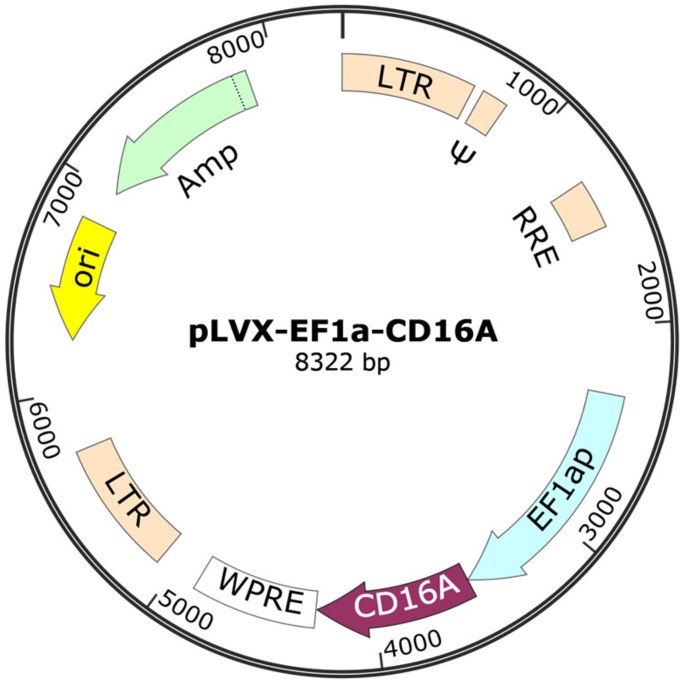

Figure S3. Plasmid map of lentivirus vector carrying human EF1a promoter-controlled CD16A gene.

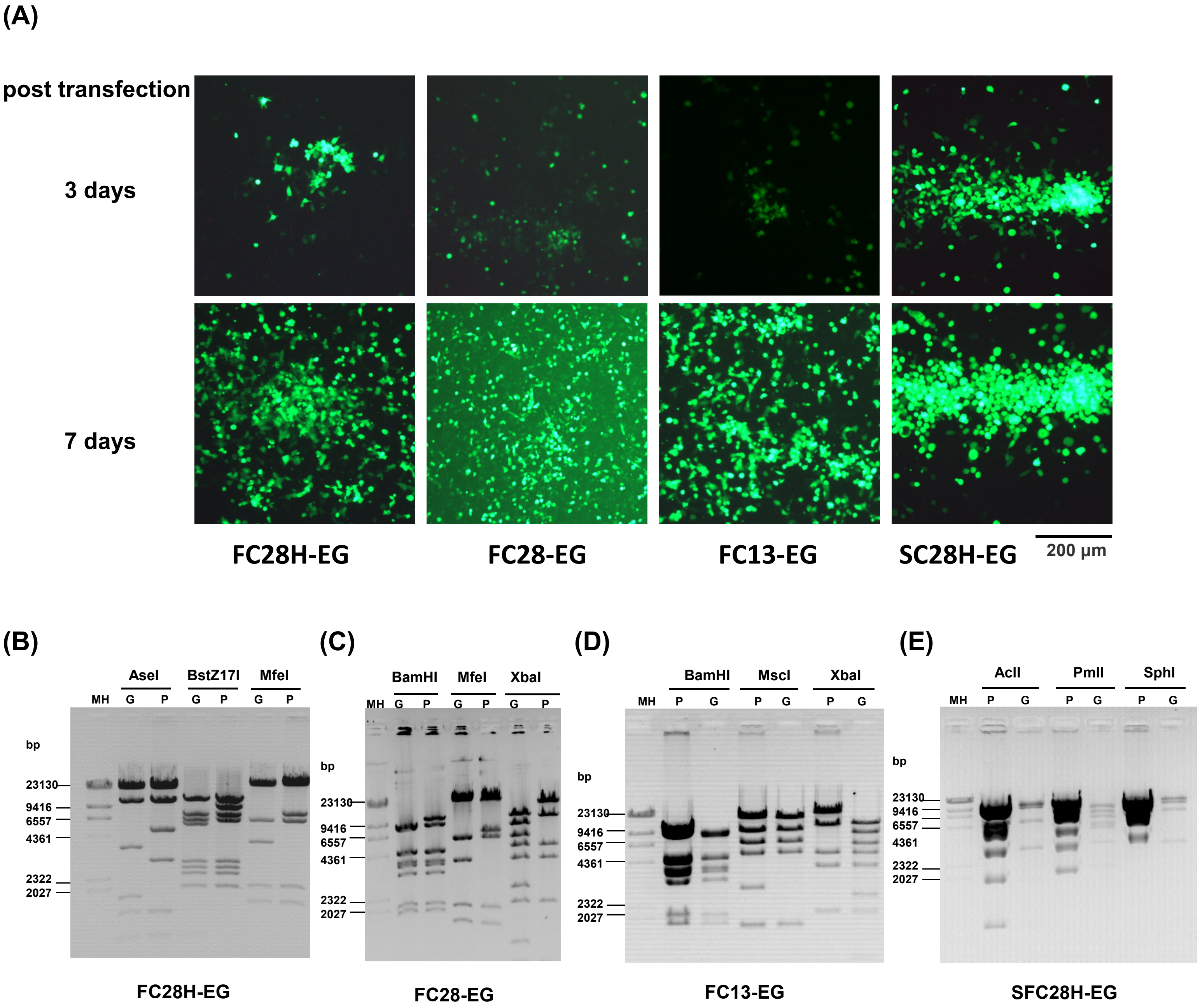

Figure S4. Rescue and identification of fiber-modified recombinant FAdV-4 viruses and SAdV-1 viruses.
(A) Rescue of fiber-modified FAdV-4 virus in PmeI-linearized the vector of fiber-modified FAdV-4 transfected LMH cells; Foci formed by GFP-positive cells were observed under a fluorescence microscope 3- or 7-days post-transfection, suggesting successful virus rescue. Rescue of fiber-modified SAdV-1 virus in SwaI-linearized the vector of fiber-modified SAdV-1 transfected SE13 cells; Foci formed by GFP-positive cells were observed under a fluorescence microscope 3- or 7-days post-transfection, suggesting successful virus rescue.
(B,C,D,F) Virus identification. **(B)** The predicted molecular weights (bp) of digested fragments of FC28H-EG genome were 21,849 bp, 11251 bp, 3537 bp, 1722 bp, 1416 bp, 988 bp, 650 bp for AseI; and 12,141 bp, 6986 bp, 6118 bp, 5557 bp, 2755 bp, 2545 bp, 2337 bp, 1956 bp, 1018 bp for BstZ17I; and 27,094 bp, 5950 bp, 3824 bp, 1983 bp, 1630 bp, 705 bp, 227 bp for MfeI; The predicted molecular weights (bp) of digested fragments of FC28H-EG plasmid were 21,849 bp, 11251 bp, 4863 bp, 2867 bp, 1416 bp, 988 bp, 650 bp for AseI; and 12,141 bp, 9046 bp, 6986 bp, 6118 bp, 2755 bp, 2545 bp, 2337 bp, 1956 bp for BstZ17I; and 27,094 bp, 7000 bp, 5950 bp, 1983 bp, 1630 bp, 227 bp for MfeI; **(C)** The predicted molecular weights (bp) of digested fragments of FC28-EG genome were 8725 bp, 8153 bp, 4550 bp, 4548 bp, 3789 bp, 3627 bp, 3140 bp, 2039 bp, 1868 bp, 531 bp, 249 bp, 105 bp, 71 bp for BamHI; and 27,094 bp, 5932 bp, 3824 bp, 1983 bp, 1630 bp, 705 bp, 227 bp for MfeI; and 11,361 bp, 8302 bp, 6008 bp, 4882 bp, 3850 bp, 2558 bp, 2123 bp, 1290 bp, 994 bp, 27 bp for XbaI; The predicted molecular weights (bp) of digested fragments of FC28-EG plasmid were 10,873 bp, 8725 bp, 4550 bp, 4548 bp, 3789 bp, 3627 bp, 3140 bp, 2039 bp, 1868 bp, 531 bp, 249 bp, 105 bp, 71 bp for BamHI; and 27,094 bp, 7000 bp, 5932 bp, 1983 bp, 1630 bp, 705 bp, 227 bp for MfeI; and 20,953 bp, 11,037 bp, 4909 bp, 3850 bp, 2123 bp, 994 bp for XbaI; **(D)** The predicted molecular weights (bp) of digested fragments of FC13-EG genome were 8725 bp, 8153 bp, 4548 bp, 4538 bp, 3789 bp, 3627 bp, 3140 bp, 2039 bp, 1868 bp, 531 bp, 249 bp, 105 bp, 71 bp for BamHI; and 15,115 bp, 9317 bp, 6424 bp, 4910 bp, 1834 bp, 1827 bp, 1059 bp, 468 bp, 429 bp for MscI; and 11,361 bp, 8302 bp, 6008 bp, 4882 bp, 3838 bp, 2558 bp, 2123 bp, 1290 bp, 994 bp, 27 bp for XbaI; The predicted molecular weights (bp) of digested fragments of FC13-EG plasmid were 10,873 bp, 8725 bp, 4548 bp, 4538 bp, 3789 bp, 3627 bp, 3140 bp, 2039 bp, 1868 bp, 531 bp, 105 bp, 71 bp for BamHI; and 15,815 bp, 9317 bp, 6424 bp, 4910 bp, 2830 bp, 1834 bp, 1827 bp, 468 bp, 429 bp for MscI; and 20,953 bp, 11,037 bp, 4909 bp, 3838 bp, 2123 bp, 994 bp for XbaI; **(E)** The predicted molecular weights (bp) of digested fragments of SFC28H-EG genome were 10,390 bp, 8666 bp, 4643 bp, 4010 bp, 2805 bp, 1235 bp, 678 bp for AclI; and 7285 bp, 5795 bp, 4047 bp, 3758 bp, 2873 bp, 2873 bp, 2105 bp, 350 bp, 177 bp for PmlI; and 11,148 bp, 8748 bp, 5734 bp, 2809 bp, 2779 bp for SphI; The predicted molecular weights (bp) of digested fragments of SFC28H-EG plasmid were 10,390 bp, 8666 bp, 4643 bp, 4010 bp, 2805 bp, 1962 bp, 1235 bp for AclI; and 11,368 bp, 7285 bp, 5795 bp, 3758 bp, 2873 bp, 2105 bp, 350 bp, 177 bp for PmlI; and 11,148 bp, 8748 bp, 5734 bp, 4883 bp, 3198 bp for SphI.

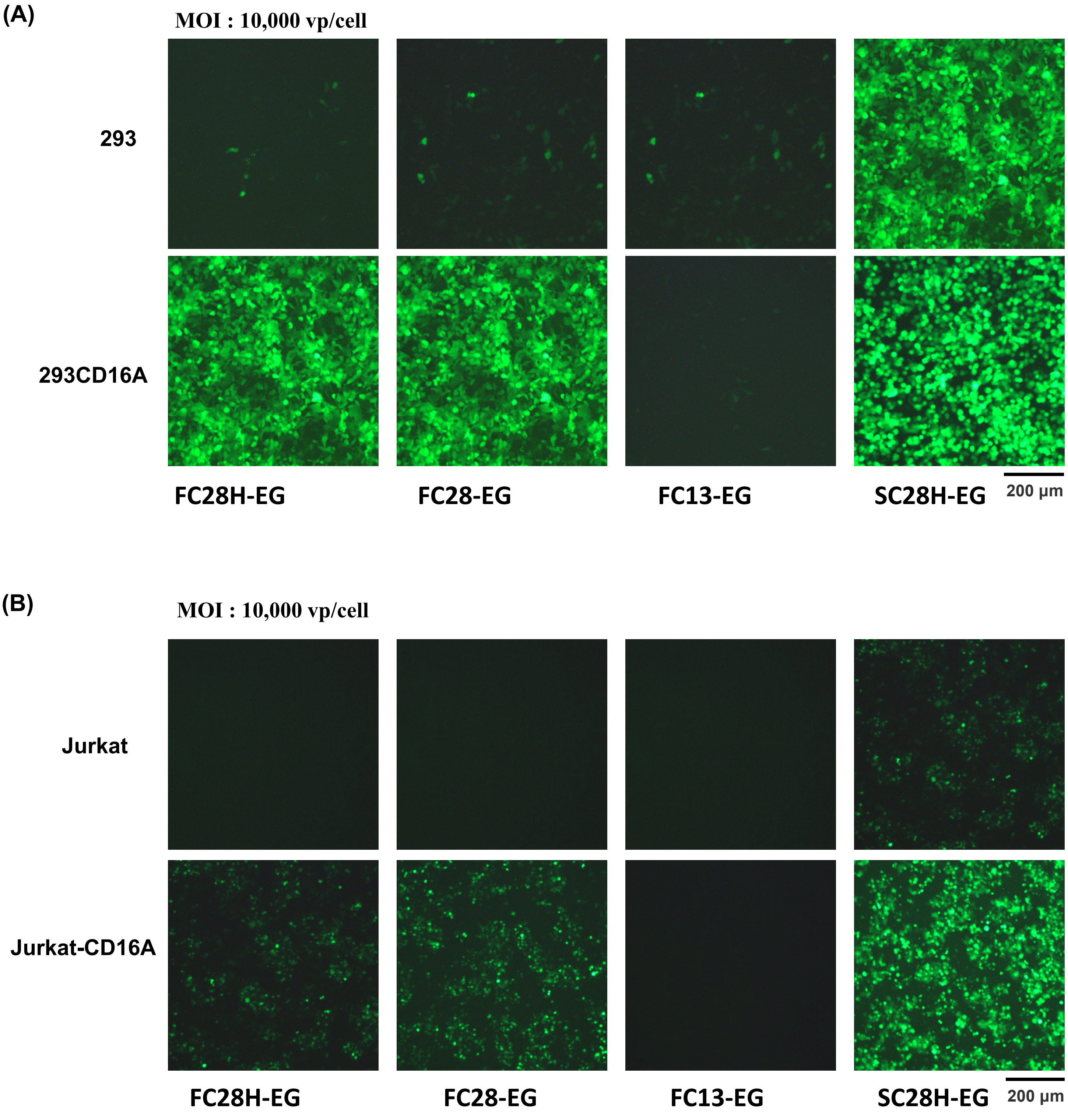

Figure S5. Adenovirus transduction experiments.
(A) 293 and 293CD16a cells were infected by the above viruses at MOI:10,000 vp/cell for 4 h. GFP expression was observed under a fluorescence microscope at 48 h post-infection.
(**B)** Jurkat and Jurkat-CD16a cells were infected by the above viruses at MOI:10,000 vp/cell for 4 h . GFP expression was observed under a fluorescence microscope at 48 h post-infection.

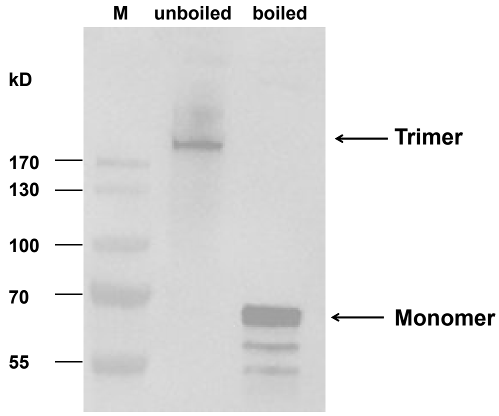

Figure S6 Detection of VHH integrated fiber in SAdV1C28H-EG by Western blot. Equal amounts (2.5×10^9 vp) of purified SAdV1C28H-EG viruses were mixed with 2 × SDS loading buffer, incubated at room temperature (unboiled) or in boiling water (boiled) for 5 minutes, loaded in SDS-PAGE gel, and transferred to nitrocellulose membranes after electrophoresis. The fiber was detected by Western blot with anti-6×His monoclonal antibody. M: protein marker. The predicted molecular weight of monomer fiber was 59.7 kD.
